# Lymph node hemophagocytosis in rickettsial diseases: a pathogenetic role for CD8 T lymphocytes in human monocytic ehrlichiosis (HME)?

**DOI:** 10.1186/1471-2334-6-121

**Published:** 2006-07-21

**Authors:** Kerry L Dierberg, J Stephen Dumler

**Affiliations:** 1 Division of Medical Microbiology, Department of Pathology, The Johns Hopkins University School of Medicine, Baltimore, Maryland, USA

## Abstract

**Background:**

Human monocytic ehrlichiosis (HME) and Rocky Mountain spotted fever (RMSF) are caused by *Ehrlichia chaffeensis *and *Rickettsia rickettsii*, respectively. The pathogenesis of RMSF relates to rickettsia-mediated vascular injury, but it is unclear in HME.

**Methods:**

To study histopathologic responses in the lymphatic system for correlates of immune injury, lymph nodes from patients with HME (n = 6) and RMSF (n = 5) were examined. H&E-stained lymph node tissues were examined for five histopathologic features, including hemophagocytosis, cellularity, necrosis, and vascular congestion and edema. The relative proportions of CD68 macrophages, CD8 and CD4 T lymphocytes, and CD20 B lymphocytes were evaluated by immunohistochemical staining.

**Results:**

Hemophagocytosis was similar in HME and RMSF, and was greater than in control cases (p = .015). Cellularity in HME was not different from controls, whereas RMSF lymph nodes were markedly less cellular (p < 0.002). *E. chaffeensis*-infected mononuclear phagocytes were infrequent compared to *R. rickettsii*-infected endothelial cells. More CD8 cells in lymph nodes were observed with HME (p < .001), but no quantitative differences in CD4 lymphocytes, macrophages, or B lymphocytes were identified.

**Conclusion:**

Hemophagocytosis, CD8 T cell expansion, and the paucity of infected cells in HME, suggest that *E. chaffeensis *infection leads to macrophage activation and immune-mediated injury.

## Background

The tick-borne obligate intracellular bacteria *Ehrlichia chaffeensis *and *Rickettsia rickettsii *are the causes of human monocytic ehrlichiosis (HME) and Rocky Mountain spotted fever (RMSF) diseases, respectively [1]. *E. chaffeensis *is transmitted by the Lone Star tick, *Amblyomma americanum*, and human infection typically presents with fever, myalgias, pancytopenia, and mild to moderate elevation of serum transaminases [2]. This clinical presentation is easily confused with that of RMSF, which typically presents with fever, rash, and headache. *R. rickettsii *is known to infect endothelial cells, leading to direct rickettsia-mediated vascular injury accompanied by a vigorous but protective Th1 immune response [3]. *E. chaffeensis *is known to infect cells in the monocyte/macrophage lineage, but the pathogenesis of HME is less clear [4]. Lymphohistiocytic-rich infiltrates, foamy macrophage infiltrates, and non-caseating granulomas suggest diffuse activation of the mononuclear phagocyte system and a role for host inflammation and immunity in its pathogenesis [5,6]. In this study, we examined documented cases of HME and RMSF in order to quantitate and further delineate the pathology and underlying pathogenesis of *E. chaffeensis *infection.

## Methods

### Patients and tissue samples

H&E-stained and unstained slides or formalin-fixed paraffin-embedded lymph node tissue blocks were obtained for 6 cases of HME (5 autopsies and 1 biopsy) and 5 representative cases of RMSF autopsies. All cases were documented by serologic studies (HME 3 cases; RMSF 1 case), PCR amplification from peripheral blood (HME 2 cases; RMSF 0 cases), or immunohistochemical demonstration of either *R. rickettsii *or *E. chaffeensis *(HME 6 cases; RMSF 5 cases). The patients ranged in age from 6 to 80 years. In addition, lymph node tissue from 8 control cases were examined, including 4 infection controls (1 case each of murine typhus [autopsy], cat scratch disease [biopsy], tuberculosis [biopsy], and gram-positive coccus lymphadenitis [biopsy]) chosen to reflect a spectrum of potential Th1 and Th2 immune induction, and 4 consecutive, random autopsy cases from the Johns Hopkins Hospital for which lymph node tissues were available at the time of study inception (random controls, including 1 patient with pulmonary embolism/cardiomyopathy, 1 patient with congestive heart failure and chronic obstructive pulmonary disease, 1 patient with a subarachnoid hemorrhage and 1 patient with myasthenia gravis and bronchopneumonia). Control cases were selected to represent a broad, but not comprehensive spectrum of potential changes observed with other infections and non-infectious processes. The lymph node specimens taken during autopsy represent hilar and/or mediastinal lymph nodes, and for biopsies, cervical or inguinal lymph nodes were sampled. Generally one or two lymph nodes per patient were evaluated and an entire 5 μm tissue section for each lymph node was reviewed for relevant histopathologic features. No lymph nodes draining skin lesions or other obvious inflammatory foci were used. Approval for study of the tissues was obtained from The Johns Hopkins Medicine Institutional Review Board.

### Histologic and immunohistologic staining

H&E-stained lymph node tissues were examined for five histopathologic features, including hemophagocytosis (macrophage activation), cellularity (inflammatory cell infiltration), necrosis (tissue damage), and vascular congestion (non-cellular tissue inflammation) and edema (vascular permeability). The relative proportions of CD68 macrophages (clone KP-1), CD8 (clone 1A5) and CD4 T lymphocytes (clone 1F6), and CD20 B lymphocytes (clone L26) were determined by immunohistochemical staining of paraffin-embedded tissues from 5 of 6 HME cases, 1 case of each of murine typhus and cat scratch disease, and 2 autopsy controls for which additional slides or tissue were available. No unstained slides or formalin-fixed paraffin-embedded lymph node tissue blocks were available for 4 of the 5 RMSF cases, prompting exclusion of RMSF from evaluation for immunophenotypic markers. All immunophenotyping antibodies were obtained from Ventana Medical Systems, Inc., (Tucson, AZ) and a modified avidin-biotin complex method was used after pretreatment of slides in citrate buffer. Specificity of each antibody reaction was confirmed prior to use consistent with the Quality Control recommendations of the College of American Pathologists Laboratory Accreditation Program, Anatomic Pathology Checklist, or the NCCLS Approved Guideline, and were confirmed with each run by using normal lymph node or tonsil tissues with defined distributions of the cell subsets studied.

Owing to the potential that semiquantitative assessment may not produce normally distributed results, we elected to use non-parametric studies to compare HME, RMSF, and control groups. This involved blinding the slides as to diagnosis and ranking the 19 cases for each histologic feature and the 9 cases for each immunophenotypic feature, respectively [7-10]. Cases were evaluated for quantity, distribution, and severity of each feature by slide-to-slide comparisons, until each case was assigned a rank number that corresponded to rank position for each feature. Ranking was conducted simultaneously by 2 microscopists to assure consensus. These ranks were evaluated for each feature using one-tailed Mann-Whitney U-statistic nonparametric tests where p values <.05 were considered significant. For each comparison of two groups (HME vs. controls, HME vs. RMSF, RMSF vs. controls), all features were re-ranked for appropriate application of Mann-Whitney U-statistical tests.

Immunohistologic demonstration of *E. chaffeensis *and *R. rickettsii *was performed using biotinylated human anti-*E. chaffeensis *for direct detection [6], *E. chaffeensis *monoclonal antibody 1A9 [11], or polyclonal rabbit anti-*R. rickettsii *[12] and appropriate biotinylated antibodies, followed by detection using either streptavidin-alkaline phosphatase (Dako) and naphthol phosphate/fast red/levamisole substrate solution or streptavidin-horseradish peroxidase and diaminobenzidine substrate. Extensive washing with phosphate-buffered saline was performed after each incubation. All specimens were counterstained with Mayer's hematoxylin, mounted with Crystal Mount (Biomeda, Hayward CA) and dried before applying coverslips. All immunohistologic preparations were examined at a magnification of 400× for identification of infected cells. *E. chaffeensis *morulae and individual *R. rickettsii *were identified by substrate reaction product, and assessed for bacterial load by a semiquantitative method ranging from none detected, graded as 0, and 1, 2, or 3 if infected cells were found in <10%, 10 to 25%, and >25% of microscopic fields, respectively.

## Results

**Figure 1:**
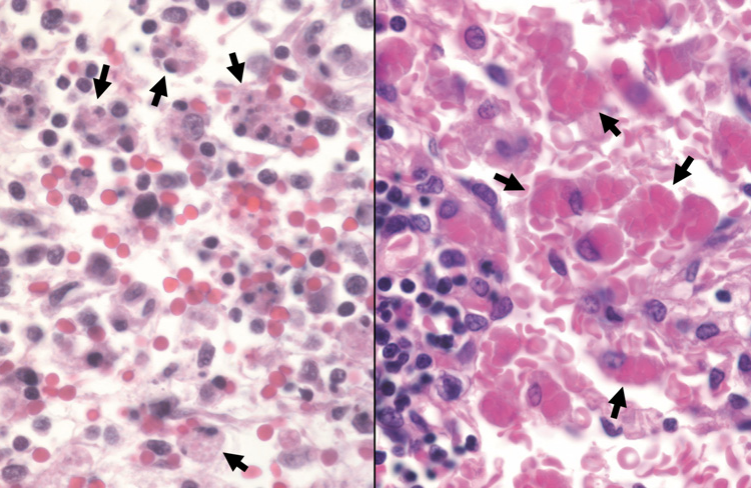
Hemophagocytosis in lymph nodes from a patient with HME (left) and RMSF (right) (H&E; original magnification 240×).

A summary of the median histopathologic rankings for HME, RMSF, and control cases for each of the five features is shown in Table 1. The main similarity between HME and RMSF was the degree of hemophagocytosis (Figure 1), with median ranks of 15 and 13 for HME and RMSF, respectively. These were significantly different from the control cases (median 3.5, p = .015 vs. HME and p = .033 vs. RMSF). CD68 macrophages were less abundant in HME than in controls. However, CD68 cells were generally detected as clusters in the lymph node medulla and paracortical regions. This feature was present to a lesser degree in one case each of RMSF, murine typhus, and cat scratch disease, but not in a lymph node from a patient with bronchopneumonia or with subarachnoid hemorrhage.

**Table 1:** Median (range) of histopathologic and immunohistologic rankings of features among patients with HME, RMSF, or controls. High ranks correspond to a greater degree for each histologic or immunohistologic feature.

**Histologic Feature n = 19**	**HME n = 6**	**RMSF n = 5**	**P HME vs. RMSF**	**Controls n = 8**	**P HME vs. controls**	**P RMSF vs. controls**
Cellularity	11.5 (1–17)	4 (2–8)	.063	13.5 (7–19)	.245	.002
Phagocytic activity	15 (3.5–19)	13 (3.5–17)	.268	3.5 (3.5–12)	.015	.033
Necrosis	10.75 (2.5–18)	10.5 (2.5–13)	.396	10.5 (3–19)	.377	.584
Congestion/edema	11.5 (2–19)	8 (2–18)	.396	11.5 (2–17)	.638	.262
Bacterial load*	1.3	3.0	.003	1.5	.447	.025

**Immunohistologic feature**	**HME [n]**		Controls [n]***		**P HME vs. controls**	

CD68 [n = 9]	7 [5] (1–10)		3.5 [4] (2–8)		.143	
CD4 [n = 8**]	5 [4**] (3–9)		6 [4] (2–8)		1.000	
CD8 [n = 9]	8 [5] (6–10)		2.5 [4] (1–5)		.008	
CD20 [n = 7**]	6 [3**] (3–8)		3.5 [4] (1–7)		.288	

* means and Student's t-tests for semiquantitative measurement of bacterial load

** immunostaining for CD4 and CD20 was unsatisfactory for 1 and 2 HME patients, respectively.

*** includes 1 case each of murine typhus, cat scratch disease, bronchopneumonia, and subarachnoid hemorrhage

**Figure 2:**
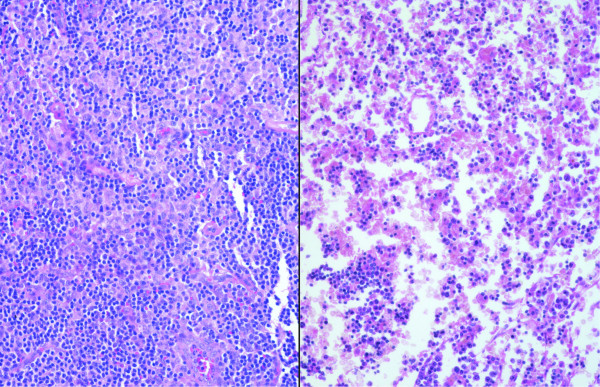
Lymph node cellularity in a patient with HME (left) and RMSF (right) (H&E; original magnification 64×).

**Figure 3:**
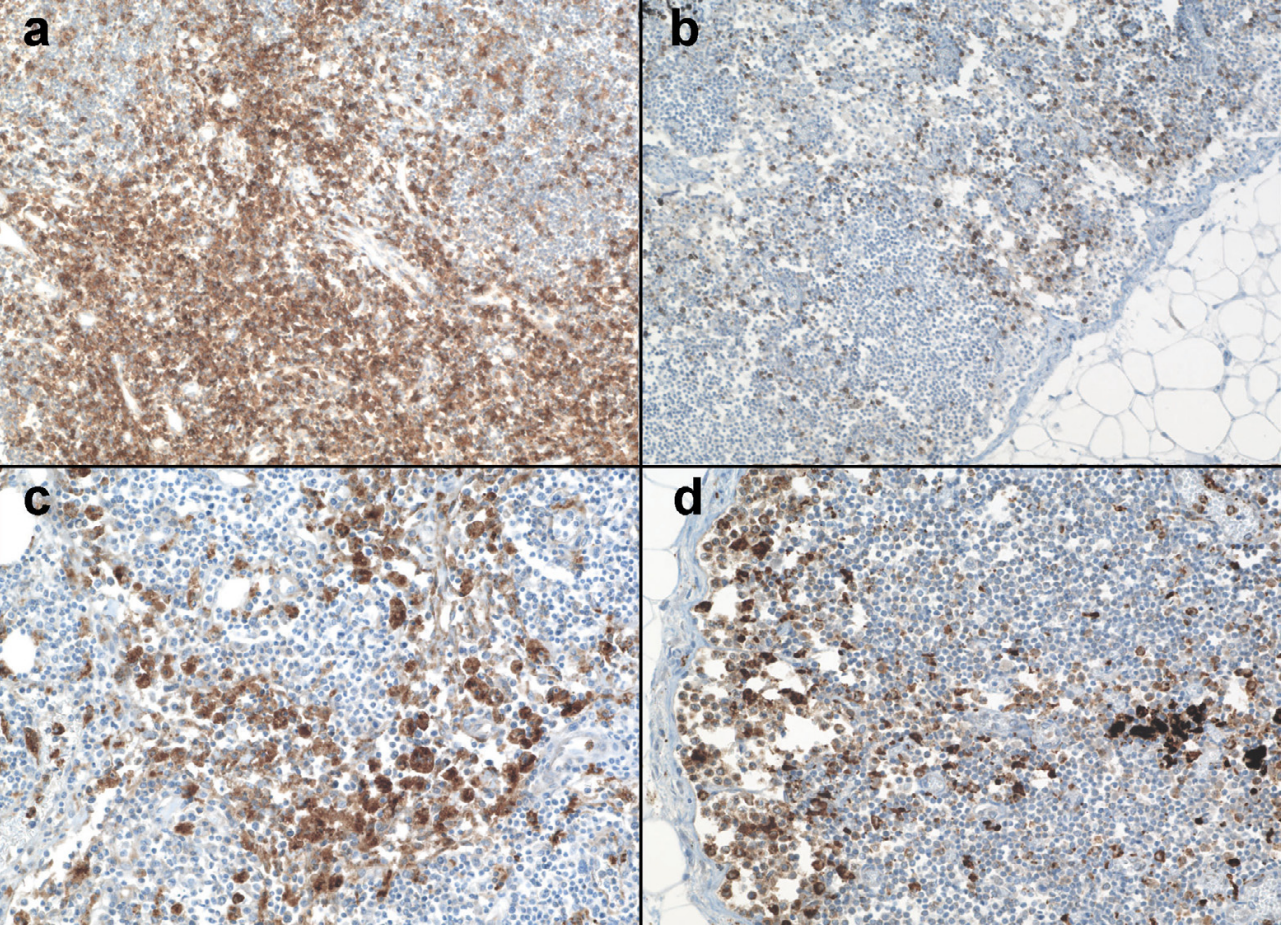
Immunophenotypic changes in lymph nodes from patients with HME. Note the marked expansion of CD8 cells in the medullary and paracortical regions (a) not observed in the control lymph node from a patient with bacterial bronchopneumonia (b). Although not quantitatively different, CD68 macrophages were more clustered within the medullary sinuses and paracortical regions in lymph nodes from HME patients (c) compared with the marginal distribution observed in a control lymph node from a patient with bacterial bronchopneumonia (d) (Immunoperoxidase with hematoxylin counterstain; original magnifications 20×).

A difference between HME (median rank 11.5) and RMSF (median rank 4) was the lower degree of cellularity found in lymph nodes of RMSF patients (Figure 2), although this difference was not significant (p = .063). In addition, lymph nodes from RMSF patients were markedly less cellular than those from controls (median rank 13.5; p = .002). Lymph nodes from patients with HME had similar degrees of cellularity as those of the infection controls (p = .245). Although variable among patients with HME, when cellularity was high, the histopathologic pattern showed proliferation of lymphocytes both in and around cortical follicles and more dominantly in the paracortical T lymphocyte regions. This pattern was also observed for the cat scratch disease control patient, whereas increased cellularity with tuberculosis included an expansion of all compartments and the additional presence of caseating and non-caseating granulomas. Overall, the lymph node histopathology in RMSF was not so much hypocellular, except as compared with the increased cellularity observed after immune stimulation with other infections, including HME. Although no differences in the degree of necrosis and congestion/edema were detected among HME or RMSF cases and controls, as anticipated, the type and pattern of necrosis were different, ranging from numerous apoptotic cells in HME and cat scratch disease, to vasculitis in RMSF and caseous necrosis in TB. Immunophenotypic evaluation of the cells in lymph nodes (Figure 3) showed no significant difference between HME cases and controls for CD68, CD4, and CD20 staining. However, HME cases demonstrated significantly more staining for CD8 than the other cases (p < .008), and these cells comprised a substantial proportion of the increased cellularity observed in the hypercellular paracortical regions of lymph nodes.

**Figure 4:**
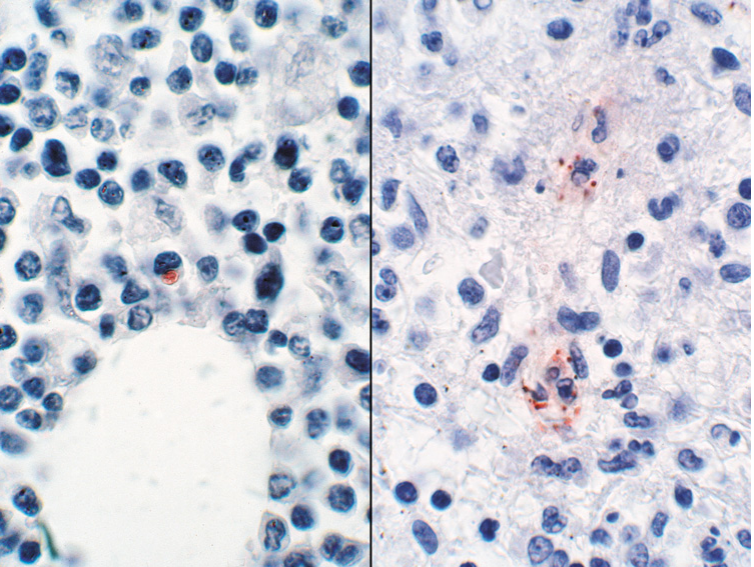
Immunohistochemical demonstration of typical bacterial burden in lymph nodes from patients with HME (left) and RMSF (right) (immunoperoxidase with hematoxylin counterstain; original magnification 240×).

Immunohistochemistry demonstrated rare to infrequent infected cells in HME compared to a significantly greater bacterial load for RMSF (mean semiquantitative grade 1.3 vs. 3.0, p = .003). *R. rickettsii *was identified at least focally in each lymph node examined from RMSF patients, whereas *E. chaffeensis *was identified only in 4 of 6 lymph nodes from HME patients. The quantity of infected cells and bacteria typical for each infection is demonstrated in Figure 4.

## Discussion

Immune-mediated control of facultative and obligate intracellular bacterial infection is particularly problematic due to the sequestered location and the lack of immune effector access to the pathogens. Thus, although many of these infections are thought to cause tissue damage by direct bacteria-mediated cell injury, an open question is to what degree host inflammation and immunity contribute to overall disease pathogenesis. Although much study of the histopathology of both HME and RMSF has been conducted, little correlation of these findings with potential pathogenetic mechanisms has been attempted; for example, the occurrence of hemophagocytosis in both entities is well described [5,13-15], but the potential importance of this feature in immunopathologic reactions is not well addressed. Tissue injury seen in RMSF is most likely a combination of direct rickettsia-mediated endothelial cell injury, manifested by vasculitis and increased vascular permeability [16,17], as well as by immune-mediated injury, demonstrated here by hemophagocytosis. Animal studies have shown that in balance, the potentially detrimental effects of Th1 immunity, local macrophage activation, and generation of cytotoxicity associated with CD8 or NK cells in rickettsial infection are beneficial to the host, owing to effective pathogen control [16,18]. IFNγ, TNFα, and other pro-inflammatory mediators produced by these cells when responding to obligate intracellular infections are known to activate macrophages, which inhibit growth of both *R. rickettsii *and *E. chaffeensis*. However, this process could also cause bystander tissue injury as suggested by the detection of hemophagocytosis [17,19]. Given the differences in bacterial load between RMSF and HME, tissue damage in HME is more likely a result of poorly controlled macrophage activation and release of effector molecules, including nitric oxide and reactive oxygen species [3,17,20].

The excessive cytokine production induced with *E. chaffeensis *infection is believed to contribute to the toxic and septic shock-like presentation seen in many cases of HME [21]. As demonstrated in this study, hemophagocytosis and evidence of inflammation-mediated tissue injury is observed in HME and RMSF to an extent greater than that in the selected infectious and non-infectious control cases. In addition, HME cases demonstrated significantly more infiltration by CD8 T lymphocytes than the control cases. This further indicates that CD8 cells and possibly cellular cytotoxicity likely play a significant role in HME pathogenesis. Whether a similar role for CD8 cells exists for RMSF, as suggested by studies in animal models, could not be adequately assessed here and needs further investigation.

Unlike the situation for the vasculotropic rickettsioses, evidence for direct ehrlichia-mediated cell injury in vivo has not been found, but the frequent presence of hemophagocytosis implicates inflammatory- and immune-mediated injury as possible major contributors. *E. chaffeensis *can cause cytolytic death of infected cells in vitro, but this is unlikely to be responsible for the degree of leukopenia, neutropenia and/or thrombocytopenia observed in patients with HME given the low frequency of infected cells in vivo as well as the lack of competent infection in platelets, neutrophils, and megakaryocytes. The relative lack of infected cells in HME compared to RMSF, despite similar degrees of hemophagocytosis, and the marked CD8 T cell expansion that could drive such responses, suggests that *Ehrlichia chaffeensis *infection often leads to macrophage activation, generation of inflammatory effectors, mediators, and cytokines, non-specific phagocytic activity, and consequent immune-mediated injury [22]. Hematologic data were not available for these cases, which in general reflect the more severe end of the disease spectrum. However, it is well recognized that patients with HME and RMSF, especially when severe, demonstrate leukopenia, thrombocytopenia, and anemia, although the disease process and degree of cytopenias differ from those observed in severe acquired or hereditary hemophagocytic syndromes [23].

The precise mechanism by which *E. chaffeensis *modulates the host innate or adaptive immunity to generate such immunologic injury is unclear. Several recent studies have begun to shed light on ehrlichial immunopathogenesis, with infection of human macrophage cell lines resulting in transcriptional alterations associated with down-regulated macrophage function [24], with production of dysfunctional cytotoxic TNFα by CD8 T lymphocytes [21], or by direct stimulation of NKT cells to produce excessive quantities of IFNγ [25].

Potential pitfalls of this study include the biased selection of patients that represent the most severe spectrum of HME or RMSF – fatal cases – and the relatively small number of cases examined. Both of these situations result from the relatively rare access to pathologically-examined cases of both HME and RMSF, limiting the power of this study. Similarly, the small number of infectious controls could equally under-represent immunopathologic responses among these entities. Despite these limitations, the small number of cases allowed at least partial study, and provides evidence of rickettsial immunopathogenicity in humans that should provide impetus for further study in both HME and RMSF, even among mild to moderately severe infections.

## Conclusion

HME and RMSF are both caused by related obligate intracellular bacteria, and both induce hemophagocytosis in lymph nodes to a degree greater than a cohort of randomly selected normal and infectious disease controls. Animal models clearly show a direct role for pathogen-induced cytotoxic injury in vivo with *R. rickettsii*. However, the relatively low quantities of *E. chaffeensis *and the marked expansion of CD8 T cells in HME support a role for immunopathology in vivo in humans, perhaps related to the dysregulated proinflammatory cytokine generation as demonstrated in animal models. Much more study will be required to dissect the immunopathogenesis of *Ehrlichia *infections, and the studies here provide additional evidence that infection may involve cytotoxic T cell activation and proliferation that could drive excessive macrophage activation as a mechanism for generating tissue-damaging effectors in HME.

## Abbreviations

HME Human monocytic ehrlichiosis

RMSF Rocky Mountain spotted fever

NK cells Natural killer cells

IFNγ Interferon gamma

TNFα Tumor necrosis factor

## Competing interests

The author(s) declare that they have no competing interests.

## Authors' contributions

KD participated in the design of the study, examination of specimens, and analysis of data and drafted the manuscript. SD conceived of the study, participated in its design and coordination, assisted with examination of specimens and analysis of data, and helped to draft the manuscript. Both authors read and approved the final manuscript.

## Pre-publication history

The pre-publication history for this paper can be accessed here:


